# Supplementary material for: The relationship between hemoglobin and V˙O2max: A systematic review and meta-analysis
Source: PLoS One. 2023 Oct 12;18(10):e0292835. doi: 10.1371/journal.pone.0292835 (PMC10569622; doi:10.1371/journal.pone.0292835)
Supplement: S2 Fig — Bubble plot and meta-regression displaying the positive association between hematocrit and both absolute V˙O2max (A) and relative V˙O2max (B). Data for males are represented as purple bubbles, data for females are represented as green bubbles with plus sign symbols, and data for studies presenting males and females pooled (mixed) are represented as black bubbles with middle dot symbols. Each bubble represents a group from a single study and the size of bubbles represents the number of participants within each group. The solid line indicates the meta-regression line, and the dashed lines indicate the 95% prediction interval associated with the meta-regression. (DOCX) [file pone.0292835.s003.docx]

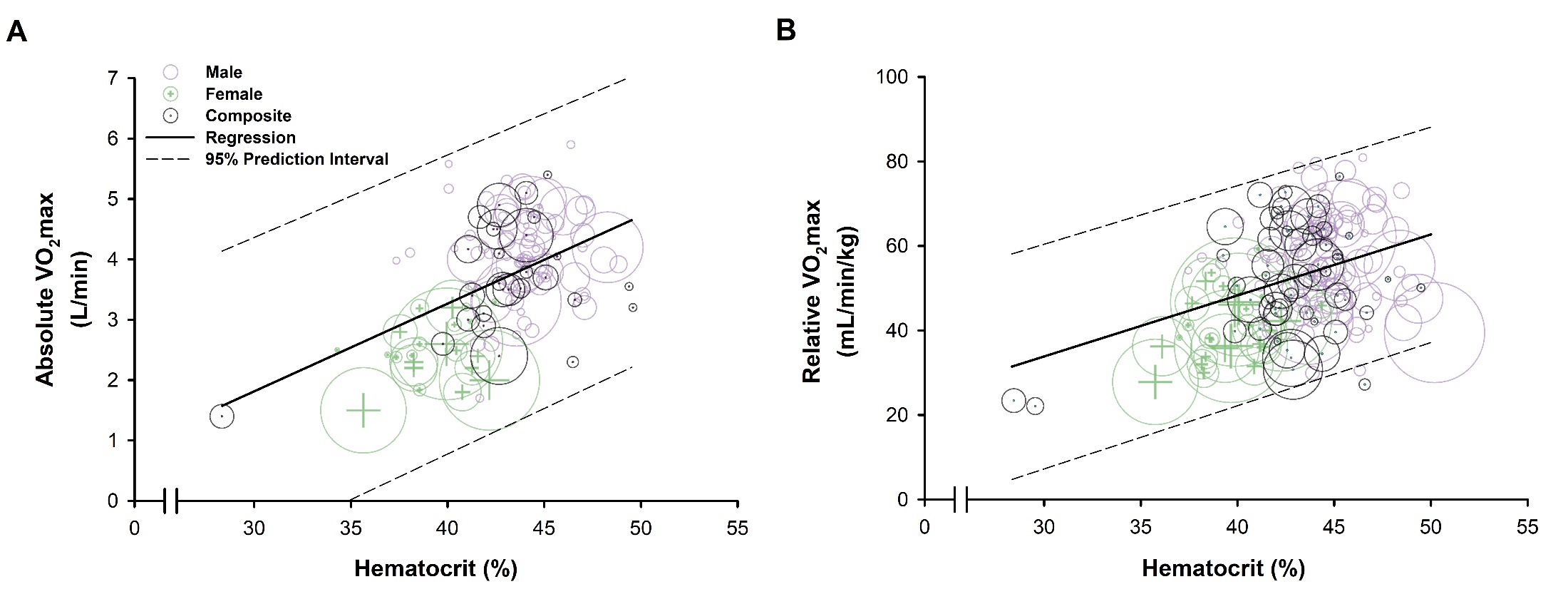
**S2 Figure.** **Association between hematocrit and maximal oxygen uptake (V̇O_2_max).** Bubble plot and meta-regression displaying the positive association between hematocrit and both absolute V̇O_2_max (A) and relative V̇O_2_max (B). Data for males are represented as purple bubbles, data for females are represented as green bubbles with plus sign symbols, and data for studies presenting males and females pooled (mixed) are represented as black bubbles with middle dot symbols. Each bubble represents a group from a single study and the size of bubbles represents the number of participants within each group. The solid line indicates the meta-regression line, and the dashed lines indicate the 95% prediction interval associated with the meta-regression.
