# Supplementary material for: The relationship between hemoglobin and V˙O2max: A systematic review and meta-analysis
Source: PLoS One. 2023 Oct 12;18(10):e0292835. doi: 10.1371/journal.pone.0292835 (PMC10569622; doi:10.1371/journal.pone.0292835)
Supplement: S1 Table — (DOCX) [file pone.0292835.s004.docx]

| **S1 Table. Risk of bias among observational articles.** | | | | | | | | |
| --- | --- | --- | --- | --- | --- | --- | --- | --- |
|  | **Risk of Bias** | | | | | | |  |
| Source | Confounding | Selection of participants | Classification of exposures | Departures from  intended exposures | Missing Data | Outcomes | Selection of  reported result | Overall Bias |
| Accalai et al. 2020 | Low | Low | Low | Low | Low | Low | Low | Low |
| Agostoni et al. 2005 | Low | **High** | Low | Low | Low | Low | Low | **High** |
| Aguiló et al. 2003 | Low | Low | Low | Low | Low | Low | Low | Low |
| Ahlgrim et al. 2009 | Low | Low | Low | Low | Low | Low | Low | Low |
| Ahmadizad et al. 2016 | Low | Low | Low | Low | Low | Low | Low | Low |
| Al Asoom et al. 2020 | Low | Low | Low | Low | Low | Low | Low | Low |
| Altehoefer et al. 2002 | Low | Low | Low | Low | Low | Low | **Moderate** | **Moderate** |
| Anchisi et al. 2001 | Low | Low | Low | Low | Low | Low | Low | Low |
| Ascha et al. 2018 | Low | Low | Low | Low | Low | Low | Low | Low |
| Awad el Karim et al. 1986 | Low | Low | Low | Low | Low | Low | Low | Low |
| Balcerek et al. 2020 | Low | Low | Low | Low | Low | Low | Low | Low |
| Barac-Nieto et al. 1978 | Low | Low | Low | Low | **Moderate** | Low | Low | **Moderate** |
| Bárány et al. 1993 | Low | Low | Low | Low | Low | Low | Low | Low |
| Baron et al. 2003 | Low | Low | Low | Low | Low | Low | Low | Low |
| Baron et al. 2008 | Low | Low | Low | Low | Low | Low | Low | Low |
| Bejder et al. 2019 | Low | Low | Low | Low | Low | Low | Low | Low |
| Bennett-Guerrero et al. 2017 | Low | Low | Low | Low | Low | Low | Low | Low |
| Berger et al. 2020 | Low | **Moderate** | Low | Low | Low | Low | Low | **Moderate** |
| Berger et al. 2021 | Low | **Moderate** | Low | Low | Low | Low | Low | **Moderate** |
| Bilé et al. 1996 | Low | Low | Low | Low | Low | Low | Low | Low |
| Böning et al. 2001 | Low | Low | Low | Low | Low | Low | Low | Low |
| Böning et al. 2004 | Low | Low | Low | Low | Low | Low | Low | Low |
| Bonner et al. 1975 | Low | Low | Low | Low | Low | Low | Low | Low |
| Bonetti et al. 2006 | Low | Low | Low | Low | Low | Low | Low | Low |
| Bouten et al. 2020 | Low | Low | Low | Low | Low | Low | Low | Low |
| Branch et al. 1997 | Low | Low | Low | Low | Low | Low | Low | Low |
| Branch et al. 1998 | Low | Low | **Moderate** | Low | Low | Low | Low | **Moderate** |
| Buick et al. 1980 | Low | Low | Low | Low | Low | Low | Low | Low |
| Burden et al 2015 | Low | Low | Low | Low | Low | Low | Low | Low |
| Burtscher et al. 2011 | Low | Low | Low | Low | Low | Low | Low | Low |
| Calbet et al. 2002 | Low | Low | Low | Low | Low | Low | Low | Low |
| Calbet et al. 2003a | Low | Low | Low | Low | Low | Low | Low | Low |
| Calbet et al. 2003b | Low | Low | Low | Low | Low | Low | Low | Low |
| Calbet et al. 2004 | Low | Low | Low | Low | Low | Low | Low | Low |
| Calbet et al. 2015 | Low | Low | Low | Low | Low | Low | Low | Low |
| Caldemeyer et al. 1996 | Low | **Moderate** | Low | Low | Low | Low | Low | **Moderate** |
| Caldwell et al. 1984 | Low | Low | Low | Low | Low | Low | Low | Low |
| Carr et al. 2015 | Low | Low | Low | Low | Low | Low | Low | Low |
| Chanda et al. 2015 | Low | Low | Low | Low | Low | Low | Low | Low |
| Ciekot-Sołtysiak et al. 2018 | Low | Low | Low | Low | Low | Low | Low | Low |
| Connes et al. 2006 | Low | Low | Low | Low | Low | Low | Low | Low |
| Córdova et al. 2019 | Low | Low | Low | Low | Low | Low | Low | Low |
| Cordova et al. 1992 | Low | Low | Low | Low | Low | Low | Low | Low |
| Costill et al. 1974 | Low | Low | Low | Low | Low | Low | Low | Low |
| Cox et al. 2004 | Low | Low | Low | Low | Low | Low | Low | Low |
| Crouter et al. 2012 | Low | Low | Low | Low | Low | Low | Low | Low |
| Davies et al. 1984 | Low | Low | Low | Low | Low | Low | Low | Low |
| Davies et al. 1973a | Low | **High** | Low | Low | Low | Low | Low | **High** |
| Davies et al. 1973b | Low | **High** | Low | Low | Low | Low | Low | **High** |
| De Bisschop et al. 2011 | Low | Low | Low | Low | Low | Low | Low | Low |
| Deitrick et al. 1991 | Low | Low | Low | Low | Low | Low | Low | Low |
| Dellavalle et al. 2012 | Low | **High** | Low | Low | Low | Low | Low | **High** |
| Dempsey et al. 1975 | Low | Low | Low | Low | Low | Low | Low | Low |
| Dengel et al. 1992 | Low | Low | Low | Low | Low | Low | Low | Low |
| Deuster et al. 1987 | Low | Low | Low | Low | Low | Low | Low | Low |
| Diaz-Canestro et al. 2021 | Low | Low | Low | Low | Low | Low | Low | Low |
| Dominelli et al. 2020 | Low | Low | Low | Low | Low | Low | Low | Low |
| Drinkwater et al. 1975 | Low | Low | Low | Low | Low | Low | **Moderate** | **Moderate** |
| Drinkwater et al. 1982 | Low | Low | Low | Low | Low | Low | Low | Low |
| Duda et al. 2003 | Low | Low | Low | Low | Low | Low | Low | Low |
| El-Sayed et al. 1996 | Low | Low | Low | Low | Low | Low | Low | Low |
| Eriksonn et al. 1971 | Low | Low | Low | Low | Low | Low | **Moderate** | **Moderate** |
| Ertl et al. 1991 | Low | Low | **Moderate** | Low | Low | Low | Low | **Moderate** |
| Falk et al. 1989 | Low | Low | Low | Low | Low | Low | Low | Low |
| Freund et al. 1995 | Low | Low | Low | Low | Low | Low | Low | Low |
| Garvican et al. 2007 | Low | Low | Low | Low | Low | Low | Low | Low |
| Gass et al. 2001 | Low | Low | Low | Low | Low | Low | Low | Low |
| Gass et al. 1983 | Low | Low | Low | Low | Low | Low | **Moderate** | **Moderate** |
| Gass et al. 1991 | Low | Low | Low | Low | Low | Low | Low | Low |
| Gatterer et al. 2021 | Low | Low | Low | Low | Low | Low | Low | Low |
| Gimenez et al. 1984 | Low | Low | Low | Low | Low | Low | Low | Low |
| Gimenez et al. 1986 | Low | Low | Low | Low | Low | Low | Low | Low |
| Gimenez et al. 1987 | Low | Low | Low | Low | Low | Low | Low | Low |
| Goodrich et al. 2018 | Low | Low | Low | Low | Low | Low | Low | Low |
| Gordon et al. 2010 | Low | Low | Low | Low | Low | Low | Low | Low |
| Gore et al. 1996 | Low | Low | **Moderate** | Low | Low | Low | Low | **Moderate** |
| Govus et al. 2017 | Low | Low | Low | Low | Low | Low | Low | Low |
| Gray et al. 1993 | Low | Low | Low | Low | Low | Low | Low | Low |
| Green et al. 1987 | Low | Low | Low | Low | Low | Low | Low | Low |
| Grover et al. 1967 | Low | **Moderate** | Low | Low | Low | Low | Low | **Moderate** |
| Gurney et al. 2021 | Low | Low | Low | Low | Low | Low | Low | Low |
| Halson et al. 2003 | Low | Low | Low | Low | Low | Low | Low | Low |
| Hausswirth et al. 1996 | Low | Low | Low | Low | Low | Low | **Moderate** | **Moderate** |
| Heaps et al. 1994 | Low | Low | Low | Low | Low | Low | Low | Low |
| Heinicke et al. 2001 | Low | Low | Low | Low | Low | Low | Low | Low |
| Heyman et al. 2020 | Low | Low | Low | Low | Low | Low | Low | Low |
| Hinghofer-Szalkay et al. 2002 | Low | Low | Low | Low | Low | Low | Low | Low |
| Holmberg et al. 2007 | Low | Low | Low | Low | Low | Low | Low | Low |
| Horvath et al. 1988 | Low | Low | Low | Low | Low | Low | Low | Low |
| Hsieh et al. 1986 | Low | Low | Low | Low | Low | Low | Low | Low |
| Hue et al. 2004 | Low | Low | Low | Low | Low | Low | Low | Low |
| Hunter et al. 2001 | Low | Low | Low | Low | **Moderate** | Low | Low | **Moderate** |
| Hutchinson et al. 1991 | Low | Low | Low | Low | Low | Low | Low | Low |
| Hutler et al. 2001 | Low | Low | Low | Low | Low | Low | Low | Low |
| Jacobs et al. 2011 | Low | Low | Low | Low | Low | Low | Low | Low |
| Jimenez et al. 1999 | Low | Low | Low | Low | Low | Low | **Moderate** | **Moderate** |
| Johnson et al. 2020 | Low | Low | Low | Low | Low | Low | Low | Low |
| Kargotich et al. 1997 | Low | Low | Low | Low | Low | Low | Low | Low |
| Kiyamu et al. 2015 | Low | Low | Low | Low | Low | Low | Low | Low |
| Koch et al. 1977 | Low | Low | Low | Low | Low | Low | Low | Low |
| Koehler et al. 2013 | Low | Low | Low | Low | Low | Low | Low | Low |
| Koivisto-Mork et al, 2021 | Low | Low | Low | Low | Low | Low | Low | Low |
| Koponen et al. 2012 | Low | Low | Low | Low | Low | Low | Low | Low |
| Lawrenson et al. 2003 | Low | **Moderate** | Low | Low | Low | Low | Low | **Moderate** |
| Lebrun et al. 1995 | Low | Low | Low | Low | Low | Low | Low | Low |
| Lespagnol et al. 2021 | Low | Low | Low | Low | Low | Low | Low | Low |
| Levine et al. 1991 | Low | Low | Low | Low | Low | Low | Low | Low |
| Lindinger et al. 1994 | Low | Low | Low | Low | Low | Low | Low | Low |
| Logan-Sprenger et al. 2015 | Low | Low | Low | Low | Low | Low | Low | Low |
| Lucía et al. 2002 | Low | Low | Low | Low | Low | Low | Low | Low |
| Lukaski et al. 2005 | Low | Low | Low | Low | Low | Low | Low | Low |
| Lundby et al. 2004 | Low | **Moderate** | Low | Low | Low | Low | Low | **Moderate** |
| Lundgren et al. 2021 | Low | Low | Low | Low | Low | Low | Low | Low |
| Lyle et al. 1992 | Low | Low | Low | Low | Low | Low | Low | Low |
| Lyons et al. 1990 | Low | Low | Low | Low | Low | Low | **Moderate** | **Moderate** |
| Mackenzie et al. 2008 | Low | Low | Low | Low | Low | Low | Low | Low |
| Magazanik et al. 1988 | Low | Low | Low | Low | Low | Low | **Moderate** | **Moderate** |
| Mairbaurl et al. 1983 | Low | Low | Low | Low | Low | Low | Low | Low |
| Mairbaurl et al. 1986 | Low | Low | Low | Low | Low | Low | Low | Low |
| Maksud et al. 1980 | Low | Low | Low | Low | Low | Low | Low | Low |
| Marconi et al., 2004 | Low | Low | Low | Low | Low | Low | Low | Low |
| Marlin et al. 2008 | Low | Low | Low | Low | Low | Low | Low | Low |
| Marrades et al., 1996 | Low | Low | Low | Low | Low | Low | Low | Low |
| Martin et al. 2019 | Low | Low | Low | Low | Low | Low | Low | Low |
| McInnis et al. 1998 | Low | Low | **High** | Low | Low | Low | Low | **High** |
| McKelvie et al. 1997 | Low | Low | Low | Low | Low | Low | Low | Low |
| McKenzie et al. 1991 | Low | Low | Low | Low | Low | Low | Low | Low |
| McMurray et al. 1983 | Low | Low | **Moderate** | Low | Low | Low | Low | **Moderate** |
| Medbø et al. 1985 | Low | Low | Low | Low | Low | Low | Low | Low |
| Metin et al. 2003 | Low | Low | Low | Low | Low | Low | Low | Low |
| Miller et al. 2005 | Low | Low | Low | Low | Low | Low | Low | Low |
| Montero et al. 2017 | Low | Low | Low | Low | Low | Low | Low | Low |
| Mora-Rodriguez et al. 2012 | Low | Low | Low | Low | Low | Low | Low | Low |
| Moraga et al. 2019 | Low | Low | Low | Low | Low | Low | Low | Low |
| Moretti et al. 2018 | Low | Low | Low | Low | Low | Low | Low | Low |
| Morgan et al. 2002 | Low | Low | Low | Low | Low | Low | Low | Low |
| Mounier et al. 2003 | Low | Low | Low | Low | Low | Low | Low | Low |
| Muza et al., 1987 | Low | Low | Low | Low | Low | Low | Low | Low |
| Nanas et al. 2009 | Low | **High** | Low | Low | Low | Low | Low | **High** |
| Novosadova et al. 1977 | Low | Low | Low | Low | Low | Low | Low | Low |
| Nuss et al. 1993 | Low | Low | Low | Low | Low | Low | Low | Low |
| O'Toole et al. 1983 | Low | Low | Low | Low | Low | Low | Low | Low |
| Oöpik et al. 2008 | Low | Low | Low | Low | Low | Low | Low | Low |
| Otsuka et al. 2021 | Low | Low | Low | Low | Low | Low | Low | Low |
| Oyono-Enguelle et al. 1999 | Low | Low | Low | Low | Low | Low | Low | Low |
| Painter et al. 2011 | Low | Low | Low | Low | Low | Low | Low | Low |
| Pastene et al. 1996 | Low | Low | Low | Low | Low | Low | Low | Low |
| Pedlar et al. 2013 | Low | Low | Low | Low | Low | Low | Low | Low |
| Peeling et al. 2009 | Low | Low | Low | Low | Low | Low | Low | Low |
| Peltonen et al. 2013 | Low | Low | Low | Low | Low | Low | Low | Low |
| Peters et al. 2000 | Low | Low | Low | Low | Low | Low | **Moderate** | **Moderate** |
| Petersen et al. 2009 | Low | Low | Low | Low | Low | Low | Low | Low |
| Petkus et al. 2019 | **Moderate** | Low | Low | Low | Low | Low | Low | **Moderate** |
| Piehl-Aulin et al. 1998 | Low | Low | Low | Low | Low | Low | Low | Low |
| Pivarnik et al. 1988 | **Moderate** | Low | Low | Low | Low | Low | Low | **Moderate** |
| Pivarnik et al. 1986 | Low | Low | Low | Low | Low | Low | Low | Low |
| Pivarnik et al. 1984 | Low | Low | Low | Low | Low | Low | Low | Low |
| Ponce-Gonzalez et al. 2015 | Low | Low | Low | Low | Low | Low | Low | Low |
| Proctor et al. 1998 | Low | Low | Low | Low | Low | Low | Low | Low |
| Prommer et al. 2007 | Low | Low | Low | Low | Low | Low | Low | Low |
| Prommer et al. 2010 | Low | Low | Low | Low | Low | Low | Low | Low |
| Ready et al. 1984 | Low | Low | Low | Low | **Moderate** | Low | Low | **Moderate** |
| Regensteiner et al. 1995 | Low | Low | Low | Low | Low | Low | Low | Low |
| Richalet et al. 1992 | Low | Low | Low | Low | Low | Low | Low | Low |
| Rietjens et al. 2002 | Low | Low | Low | Low | Low | Low | **Moderate** | **Moderate** |
| Robergs et al. 1998 | Low | Low | Low | Low | Low | Low | Low | Low |
| Robertson et al. 2010 | Low | Low | Low | Low | Low | Low | Low | Low |
| Robinson et al. 1995 | Low | Low | Low | Low | Low | Low | Low | Low |
| Robinson et al. 2007 | Low | Low | Low | Low | Low | Low | Low | Low |
| Rotstein et al. 1998 | Low | Low | Low | Low | Low | Low | Low | Low |
| Roy et al. 2006 | **Moderate** | Low | Low | Low | Low | Low | Low | **Moderate** |
| Sara et al. 2003 | Low | Low | Low | Low | Low | Low | Low | Low |
| Sara et al. 2006a | Low | Low | Low | Low | Low | Low | Low | Low |
| Sara et al. 2006b | Low | Low | Low | Low | Low | Low | Low | Low |
| Saunders et al. 2004 | Low | Low | Low | Low | Low | Low | Low | Low |
| Sawka et al. 1984 | Low | Low | Low | Low | Low | Low | Low | Low |
| Schierbauer et al. 2021 | Low | Low | Low | Low | Low | Low | Low | Low |
| Schleh et al. 2018 | Low | Low | Low | Low | Low | Low | Low | Low |
| Schmidt et al. 1991 | Low | Low | Low | Low | Low | Low | Low | Low |
| Schmidt et al. 2002 | Low | Low | Low | Low | Low | Low | Low | Low |
| Schmidt et al. 2020 | Low | Low | Low | Low | Low | Low | Low | Low |
| Schobersberger et al. 1990 | Low | Low | Low | Low | Low | Low | Low | Low |
| Schommer et al. 2010 | Low | Low | Low | Low | Low | Low | Low | Low |
| Schumacher et al. 2002 | Low | Low | Low | Low | Low | Low | Low | Low |
| Schumacher et al. 2008 | Low | Low | Low | Low | Low | Low | Low | Low |
| Schumacher et al. 2009 | Low | Low | Low | Low | Low | Low | Low | Low |
| Shizukuda et al. 2012 | Low | Low | Low | Low | Low | Low | Low | Low |
| Smith et al. 2006 | Low | Low | Low | Low | Low | Low | Low | Low |
| Sohn et al. 2013 | Low | **High** | Low | Low | Low | Low | Low | **High** |
| Sperlich et al. 2015 | Low | Low | Low | Low | Low | Low | Low | Low |
| Spodaryk et al. 1993 | Low | Low | Low | Low | Low | Low | Low | Low |
| Staessen et al. 1987 | Low | Low | Low | Low | Low | Low | Low | Low |
| Steiner et al. 2011 | Low | Low | Low | Low | Low | Low | Low | Low |
| Steiner et al. 2019 | Low | Low | Low | Low | Low | Low | Low | Low |
| Stephenson et al. 1988 | Low | Low | Low | Low | Low | Low | Low | Low |
| Stephenson et al. 1989 | Low | Low | Low | Low | Low | Low | Low | Low |
| Stevenson et al. 1994 | **Moderate** | Low | Low | Low | Low | Low | Low | **Moderate** |
| Stewart et al. 2020 | Low | Low | Low | Low | Low | Low | Low | Low |
| Stewart et al., 2021 | Low | Low | Low | Low | Low | Low | Low | Low |
| Stringer et al. 1997 | Low | Low | Low | Low | Low | Low | Low | Low |
| Svendsen et al. 2016 | Low | Low | Low | Low | Low | Low | Low | Low |
| Tagougui et al. 2015 | Low | Low | Low | Low | Low | Low | Low | Low |
| Telford et al. 2003 | Low | Low | Low | Low | Low | Low | Low | Low |
| Tomkiewicz-Pajak et al. 2014 | Low | Low | Low | Low | Low | Low | Low | Low |
| Treff et al. 2014 | Low | Low | Low | Low | Low | Low | Low | Low |
| Trikas et al. 1998 | Low | **High** | Low | Low | Low | Low | Low | **High** |
| Upton et al. 1984 | Low | Low | Low | Low | Low | Low | Low | Low |
| Van der Zwaard et al. 2018 | Low | Low | Low | Low | Low | Low | Low | Low |
| Vasileiadis et al. 2009 | Low | **High** | Low | Low | Low | Low | Low | **High** |
| Vistisen et al. 2003 | Low | Low | Low | Low | Low | Low | Low | Low |
| Vogt et al. 2008 | Low | Low | Low | Low | Low | Low | Low | Low |
| Walker et al. 2020 | Low | Low | Low | Low | Low | Low | Low | Low |
| Warrington et al. 2001 | Low | Low | Low | Low | Low | Low | Low | Low |
| Wasserman et al. 1997 | Low | Low | Low | Low | Low | Low | Low | Low |
| Wenger et al. 1992 | Low | Low | Low | Low | Low | Low | Low | Low |
| Wetter et al. 2021 | Low | Low | Low | Low | Low | Low | Low | Low |
| Wiebe et al. 1998 | Low | Low | Low | Low | Low | Low | Low | Low |
| Wilkerson et al. 1977 | Low | Low | Low | Low | Low | Low | Low | Low |
| Wilkerson et al. 1982 | Low | Low | Low | Low | Low | Low | Low | Low |
| Witte et al. 2004 | Low | Low | Low | Low | Low | Low | Low | Low |
| Wolfel et al. 1991 | Low | Low | Low | Low | Low | Low | Low | Low |
| Wu et al. 1996 | Low | Low | Low | Low | Low | Low | Low | Low |
| Yaspelkis et al. 1991 | Low | Low | Low | Low | Low | Low | **Moderate** | **Moderate** |
| Zavorsky et al. 2005 | Low | Low | Low | Low | Low | Low | Low | Low |
| Zheng et al. 2017 | Low | Low | Low | Low | Low | Low | Low | Low |
| Zhu & Haas, 1997 | Low | **High** | Low | Low | Low | Low | Low | **High** |
