# Supplementary material for: The relationship between hemoglobin and V˙O2max: A systematic review and meta-analysis
Source: PLoS One. 2023 Oct 12;18(10):e0292835. doi: 10.1371/journal.pone.0292835 (PMC10569622; doi:10.1371/journal.pone.0292835)
Supplement: S2 Table — (DOCX) [file pone.0292835.s005.docx]

| **S2 Table. Risk of bias among interventional articles.** | | | | | | | | |
| --- | --- | --- | --- | --- | --- | --- | --- | --- |
|  | Risk of Bias |  |  |  |  |  |  |  |
| Source | Confounding | Selection of participants | Classification of  exposures | Departures from  intended exposures | Missing Data | Outcomes | Selection of  reported result | **Overall Bias** |
| Ade et al. 2017 | Low | Low | Low | Low | Low | Low | Low | **Low** |
| Annaheim et al. 2016 | Low | Low | Low | Low | Low | Low | Low | **Low** |
| Aoyagi et al. 1994 | Low | Low | Low | Low | Low | Low | Low | **Low** |
| Audran et al. 1999 | Low | Low | **Moderate** | Low | Low | Low | Low | **Moderate** |
| Balke et al. 1954 | Low | Low | Low | Low | Low | Low | Low | **Low** |
| Balsom et al. 1994 | Low | Low | Low | Low | Low | Low | Low | **Low** |
| Basset et al. 2006 | Low | Low | Low | Low | Low | Low | Low | **Low** |
| Baumann et al. 2014 | Low | Low | Low | Low | Low | Low | Low | **Low** |
| Ben Abderrahman et al. 2013 | Low | Low | Low | Low | Low | Low | Low | **Low** |
| Bender et al. 1988 | Low | Low | Low | Low | Low | Low | Low | **Low** |
| Benedetto et al. 2015 | Low | **High** | Low | Low | Low | Low | Low | **High** |
| Berger et al. 2006 | Low | Low | Low | Low | Low | Low | Low | **Low** |
| Birkeland et al. 2000 | Low | Low | Low | Low | Low | Low | Low | **Low** |
| Birnbaum et al. 2006 | Low | Low | Low | Low | Low | Low | Low | **Low** |
| Bonne et al. 2014a | Low | Low | Low | Low | Low | Low | Low | **Low** |
| Bonne et al. 2014b | Low | Low | Low | Low | **Moderate** | Low | Low | **Moderate** |
| Bourque et al. 1997 | Low | Low | **Moderate** | Low | Low | Low | Low | **Moderate** |
| Boutellier et al. 1990 | Low | Low | Low | Low | Low | Low | Low | **Low** |
| Branch et al. 1999 | Low | Low | Low | Low | Low | Low | Low | **Low** |
| Bringard et al. 2010 | Low | Low | Low | Low | Low | Low | Low | **Low** |
| Brownlie et al. 2002 | Low | **Moderate** | Low | Low | Low | Low | Low | **Moderate** |
| Brugniaux et al. 2006 | Low | Low | Low | Low | Low | Low | Low | **Low** |
| Burnley et al. 2006 | Low | Low | Low | Low | Low | Low | Low | **Low** |
| Caillaud et al. 2015 | Low | Low | Low | Low | Low | Low | Low | **Low** |
| Cardinale et al. 2019 | Low | Low | Low | Low | Low | Low | Low | **Low** |
| Celsing et al. 1986a | Low | Low | Low | Low | Low | Low | Low | **Low** |
| Celsing et al. 1986b | Low | Low | Low | Low | Low | Low | Low | **Low** |
| Celsing et al. 1987 | Low | Low | Low | Low | Low | Low | Low | **Low** |
| Chapman et al. 1998 | Low | Low | Low | Low | Low | Low | Low | **Low** |
| Chen et al. 2014 | Low | Low | Low | Low | Low | Low | Low | **Low** |
| Cheng et al. 2019 | Low | Low | Low | Low | Low | Low | **Moderate** | **Moderate** |
| Christensen et al. 2013 | Low | Low | Low | Low | Low | Low | Low | **Low** |
| Clark et al. 2004 | Low | Low | Low | Low | Low | Low | Low | **Low** |
| Connes et al. 2003 | Low | Low | Low | Low | Low | Low | Low | **Low** |
| Convertino et al. 1980 | Low | Low | Low | Low | Low | Low | Low | **Low** |
| Coppola et al. 2004 | Low | **Moderate** | Low | Low | Low | Low | Low | **Moderate** |
| Costa et al. 2019 | Low | Low | Low | Low | Low | Low | Low | **Low** |
| Coyle et al. 1990 | Low | Low | Low | Low | Low | Low | Low | **Low** |
| Cureton et al. 1986 | Low | Low | Low | Low | Low | Low | Low | **Low** |
| Czuba et al. 2011 | Low | Low | Low | Low | Low | Low | Low | **Low** |
| Czuba et al. 2014 | Low | Low | Low | Low | Low | Low | Low | **Low** |
| Dehnert et al. 2002 | Low | Low | Low | Low | Low | **Moderate** | Low | **Moderate** |
| Dias et al. 2019 | Low | Low | Low | Low | Low | Low | Low | **Low** |
| Dressendorfer et al. 1991 | Low | Low | Low | **Moderate** | Low | Low | Low | **Moderate** |
| Durkalec-Michalski et al. 2021 | Low | Low | Low | Low | Low | Low | Low | **Low** |
| Durussel et al. 2013 | Low | Low | Low | Low | Low | Low | Low | **Low** |
| Eastwood et al. 2012a | Low | Low | Low | Low | Low | Low | Low | **Low** |
| Eastwood et al. 2012b | Low | Low | Low | Low | Low | Low | Low | **Low** |
| Eliassen et al. 2018 | Low | Low | Low | Low | Low | Low | Low | **Low** |
| Farzad et al. 2011 | Low | Low | Low | Low | Low | Low | Low | **Low** |
| Fernandez et al. 2019 | Low | Low | Low | Low | Low | Low | Low | **Low** |
| Ferretti et al. 1997 | Low | Low | Low | Low | **Moderate** | Low | Low | **Moderate** |
| Freedson et al. 1981 | Low | Low | Low | Low | Low | Low | Low | **Low** |
| Friman et al. 1979 | Low | Low | Low | Low | Low | Low | Low | **Low** |
| Garver et al. 2018 | Low | Low | Low | Low | Low | Low | Low | **Low** |
| Garvican et al. 2014 | **Moderate** | Low | Low | Low | Low | Low | Low | **Moderate** |
| Goodman et al. 1989 | Low | Low | Low | Low | Low | Low | Low | **Low** |
| Gordon et al. 2014 | Low | Low | Low | Low | Low | Low | Low | **Low** |
| Grassi et al. 1996 | Low | Low | Low | Low | Low | Low | Low | **Low** |
| Green et al. 1991 | Low | Low | Low | Low | Low | Low | Low | **Low** |
| Green et al. 2000 | Low | Low | Low | Low | Low | Low | Low | **Low** |
| Guadalupe-Grau et al. 2015 | Low | Low | Low | Low | Low | Low | Low | **Low** |
| Hahn et al. 2001 | Low | Low | Low | Low | **Moderate** | Low | Low | **Moderate** |
| Haider et al. 2020 | Low | Low | Low | Low | Low | Low | Low | **Low** |
| Haile et al. 2019 | Low | Low | Low | **Moderate** | Low | Low | Low | **Moderate** |
| Heuberger et al., 2017 | Low | Low | Low | Low | Low | Low | Low | **Low** |
| Hill et al., 2013 | Low | Low | Low | Low | Low | Low | Low | **Low** |
| Hinton et al. 2000 | Low | Low | Low | Low | Low | Low | Low | **Low** |
| Hinton et al. 2007 | Low | Low | Low | Low | Low | Low | Low | **Low** |
| Ingjer et al., 1992 | Low | Low | Low | Low | Low | Low | **Moderate** | **Moderate** |
| Jacobs et al. 2013 | Low | Low | Low | Low | Low | Low | Low | **Low** |
| Kanstrup et al. 1984 | Low | Low | Low | Low | Low | Low | Low | **Low** |
| Kanstrup et al. 1982 | Low | Low | Low | Low | Low | Low | Low | **Low** |
| Kilbom et al., 1971 | Low | Low | Low | Low | Low | Low | Low | **Low** |
| Klimek et al., 2010 | Low | Low | Low | Low | Low | Low | Low | **Low** |
| Klingshirn et al., 1992 | Low | Low | Low | Low | Low | Low | Low | **Low** |
| Koivisto et al., 2018 | Low | Low | Low | Low | Low | Low | Low | **Low** |
| Koskolou et al., 1997 | Low | Low | Low | Low | Low | Low | Low | **Low** |
| Kreider et al., 1990 | Low | Low | Low | Low | Low | Low | Low | **Low** |
| LaManca et al., 1993 | Low | Low | Low | Low | Low | Low | Low | **Low** |
| Lukaski et al. 1991 | Low | Low | Low | Low | Low | Low | Low | **Low** |
| Lundby et al. 2005 | Low | Low | Low | Low | Low | Low | Low | **Low** |
| Lundby et al. 2008a | Low | Low | Low | Low | Low | Low | Low | **Low** |
| Lundby et al. 2008b | Low | Low | Low | Low | Low | Low | Low | **Low** |
| Magazanik et al. 1991 | Low | Low | Low | Low | Low | Low | Low | **Low** |
| Maldonado-Martin et al. 2017 | Low | Low | Low | Low | Low | Low | Low | **Low** |
| Marley et al. 2020 | Low | Low | Low | Low | Low | Low | Low | **Low** |
| Maron et al. 1988 | Low | Low | Low | Low | Low | Low | Low | **Low** |
| McDonagh et al. 2016 | Low | Low | Low | Low | Low | Low | Low | **Low** |
| McMurray et al. 1985 | Low | Low | Low | Low | Low | Low | Low | **Low** |
| Menz et al. 2015 | Low | Low | Low | Low | Low | Low | Low | **Low** |
| Meurrens et al. 2016 | Low | Low | Low | Low | Low | Low | **Moderate** | **Moderate** |
| Mier et al. 1996 | Low | Low | Low | Low | Low | Low | Low | **Low** |
| Montero et al. 2015 | Low | Low | Low | Low | Low | Low | Low | **Low** |
| Moro et al. 2020 | Low | Low | Low | Low | Low | Low | Low | **Low** |
| Morton et al. 2005 | Low | Low | Low | Low | Low | Low | Low | **Low** |
| Mounier et al. 2006 | Low | Low | **Moderate** | Low | Low | Low | Low | **Moderate** |
| Nakamoto et al. 2016 | Low | Low | Low | Low | **Moderate** | Low | Low | **Moderate** |
| Newhouse et al. 1989 | Low | Low | Low | Low | Low | Low | Low | **Low** |
| Nordsborg et al. 2015 | Low | Low | Low | Low | Low | Low | Low | **Low** |
| Okazaki et al. 2019 | Low | Low | Low | Low | Low | Low | Low | **Low** |
| Panebianco et al. 1995 | Low | Low | Low | Low | Low | Low | Low | **Low** |
| Park et al. 2019 | Low | Low | Low | Low | Low | Low | Low | **Low** |
| Peeling et al. 2007 | Low | Low | Low | Low | Low | Low | Low | **Low** |
| Podgorski et al. 2015 | Low | Low | Low | Low | Low | Low | Low | **Low** |
| Pompano et al. 2017 | Low | Low | Low | Low | Low | Low | Low | **Low** |
| Puhl et al. 1980 | Low | Low | Low | Low | Low | Low | Low | **Low** |
| Putman et al. 2003 | Low | Low | Low | Low | Low | Low | Low | **Low** |
| Ramos-Campo et al. 2015 | Low | Low | Low | Low | Low | Low | Low | **Low** |
| Ramos-Campo et al. 2018 | Low | Low | Low | Low | Low | Low | Low | **Low** |
| Rasic et al. 2021 | Low | Low | Low | Low | Low | Low | Low | **Low** |
| Rietjens et al. 2005 | Low | Low | Low | Low | Low | Low | Low | **Low** |
| Robach et al. 2006b | Low | Low | Low | Low | Low | Low | Low | **Low** |
| Robach et al. 2008 | Low | Low | Low | Low | Low | Low | Low | **Low** |
| Robach et al. 2012 | Low | Low | Low | Low | Low | Low | Low | **Low** |
| Robach et al. 2014 | Low | Low | Low | Low | Low | Low | Low | **Low** |
| Robach et al. 2018 | Low | Low | Low | Low | Low | Low | Low | **Low** |
| Robertson et al. 2010b | Low | Low | Low | Low | Low | Low | Low | **Low** |
| Robertson et al. 1982 | Low | Low | Low | Low | Low | Low | Low | **Low** |
| Robertson et al. 1984 | Low | Low | Low | Low | Low | Low | **Moderate** | **Moderate** |
| Roels et al. 2005 | Low | Low | Low | Low | Low | Low | Low | **Low** |
| Rønnestad et al. 2014 | Low | Low | Low | Low | Low | Low | Low | **Low** |
| Rønnestad et al. 2021 | Low | Low | Low | Low | Low | Low | Low | **Low** |
| Ryan et al. 2016 | Low | Low | Low | Low | Low | Low | Low | **Low** |
| Saugy et al. 2016 | Low | Low | Low | Low | Low | Low | Low | **Low** |
| Saunders et al. 2009 | Low | Low | Low | Low | Low | Low | Low | **Low** |
| Schaffartzik et al 1993 | Low | Low | Low | Low | Low | Low | Low | **Low** |
| Schmidt et al. 1988 | Low | Low | Low | Low | Low | Low | **Moderate** | **Moderate** |
| Schoene et al. 1983 | Low | Low | Low | Low | Low | Low | Low | **Low** |
| Sheykhlouvand et al. 2016 | Low | Low | Low | Low | Low | Low | Low | **Low** |
| Shoemaker et al. 1996 | Low | Low | Low | Low | Low | Low | Low | **Low** |
| Sieljacks et al. 2016 | Low | Low | Low | Low | Low | Low | Low | **Low** |
| Sitkowski et al. 2021 | Low | Low | Low | Low | Low | Low | Low | **Low** |
| Skattebo et al. 2020 | Low | Low | Low | Low | **Moderate** | Low | Low | **Moderate** |
| Skattebo et al. 2021 | Low | Low | Low | Low | Low | Low | Low | **Low** |
| Sotiridis et al. 2018 | Low | Low | Low | Low | Low | Low | Low | **Low** |
| Spriet et al. 1986 | Low | Low | Low | Low | Low | Low | Low | **Low** |
| Steinacker et al. 1996 | Low | Low | Low | Low | Low | Low | Low | **Low** |
| Stevenson et al. 2016 | Low | Low | Low | Low | Low | Low | Low | **Low** |
| Stray-Gundersen et al. 2001 | Low | Low | Low | Low | Low | Low | Low | **Low** |
| Svedenhag et al. 1997 | Low | Low | Low | Low | Low | Low | Low | **Low** |
| Taniguchi et al. 1991 | Low | Low | Low | Low | Low | Low | Low | **Low** |
| Taylor et al. 1997 | Low | Low | Low | Low | Low | Low | Low | **Low** |
| Thomsen et al. 2007 | Low | Low | Low | Low | Low | Low | Low | **Low** |
| Turner et al. 1993 | Low | Low | Low | Low | Low | Low | Low | **Low** |
| Veicsteinas et al. 1984 | Low | Low | **High** | Low | Low | Low | Low | **High** |
| Vukovich et al. 2001 | Low | Low | Low | Low | Low | Low | Low | **Low** |
| Wachsmuth et al. 2015 | Low | Low | Low | Low | Low | Low | Low | **Low** |
| Wang et al. 2019 | Low | Low | Low | Low | Low | Low | Low | **Low** |
| Warburton et al. 1999 | Low | Low | Low | Low | Low | Low | Low | **Low** |
| Wehrlin et al. 2006 | Low | Low | Low | Low | Low | Low | Low | **Low** |
| Wirth et al. 1978 | Low | Low | Low | Low | **Moderate** | Low | Low | **Moderate** |
| Woodson et al. 1978 | Low | Low | Low | Low | Low | Low | Low | **Low** |
| Yan et al. 2021 | Low | Low | Low | Low | Low | Low | Low | **Low** |
| Zapico et al. 2007 | Low | Low | Low | Low | Low | Low | Low | **Low** |
| Ziegler et al. 2015 | Low | Low | Low | Low | Low | Low | Low | **Low** |
| Zorbas et al. 1997 | Low | Low | Low | Low | Low | Low | Low | **Low** |
| Zorbas et al. 1998 | Low | Low | Low | Low | Low | Low | Low | **Low** |
