# Supplementary material for: The relationship between hemoglobin and V˙O2max: A systematic review and meta-analysis
Source: PLoS One. 2023 Oct 12;18(10):e0292835. doi: 10.1371/journal.pone.0292835 (PMC10569622; doi:10.1371/journal.pone.0292835)
Supplement: Attachment — Submitted filename: PONE-D-22-29015-R2R1.docx [file pone.0292835.s007.docx]

**Academic Editor**

Thank you for submitting your manuscript to PLOS ONE. After careful consideration, we feel that it has merit but does not fully meet PLOS ONE’s publication criteria as it currently stands. Therefore, we invite you to submit a revised version of the manuscript that addresses the points raised during the review process.

**Response:** Thank you for the opportunity to revise this manuscript and respond to comments from expert reviewers. The comments from the reviewers enhanced the quality and potential impact of this manuscript, and the comments have been responded to accordingly.

**Journal Requirements:**

When submitting your revision, we need you to address these additional requirements.

**1.** Please ensure that your manuscript meets PLOS ONE's style requirements, including those for file naming. The PLOS ONE style templates can be found at

<https://journals.plos.org/plosone/s/file?id=wjVg/PLOSOne_formatting_sample_main_body.pdf> and

[https://journals.plos.org/plosone/s/file?id=ba62/PLOSOne_formatting_sample_title_authors_affiliations.pdf](https://nam12.safelinks.protection.outlook.com/?url=https%3A%2F%2Fjournals.plos.org%2Fplosone%2Fs%2Ffile%3Fid%3Dba62%2FPLOSOne_formatting_sample_title_authors_affiliations.pdf&data=05%7C01%7CWebb.Kevin%40mayo.edu%7C3670d02a5e2a47acaae208dae7a4a039%7Ca25fff9c3f634fb29a8ad9bdd0321f9a%7C0%7C0%7C638076991516309025%7CUnknown%7CTWFpbGZsb3d8eyJWIjoiMC4wLjAwMDAiLCJQIjoiV2luMzIiLCJBTiI6Ik1haWwiLCJXVCI6Mn0%3D%7C3000%7C%7C%7C&sdata=ub7nR165Qw3OsRdHL501vYYzmRVmegMkZwr3Ye33PvQ%3D&reserved=0)

**Response:** Done. We have ensured that the revised manuscript meets applicable journal requirements.

**2.** Thank you for stating the following financial disclosure:

“The funders had no role in study design, data collection and analysis, decision to publish, or preparation of the manuscript.”

At this time, please address the following queries:

**a)** Please clarify the sources of funding (financial or material support) for your study. List the grants or organizations that supported your study, including funding received from your institution.

**b)** State what role the funders took in the study. If the funders had no role in your study, please state: “The funders had no role in study design, data collection and analysis, decision to publish, or preparation of the manuscript.”

**c)** If any authors received a salary from any of your funders, please state which authors and which funders.

**d)** If you did not receive any funding for this study, please state: “The authors received no specific funding for this work.”

Please include your amended statements within your cover letter; we will change the online submission form on your behalf.

**Response:** Thank you for highlighting this omission. This work was supported by the National Heart, Lung, and Blood Institute T-32-HL105355 (K.L.W.), R-35-HL139854 (C.C.W. and M.J.J.), and F-32-HL131151 (J.W.S.).

**3.** In your Data Availability statement, you have not specified where the minimal data set underlying the results described in your manuscript can be found. PLOS defines a study's minimal data set as the underlying data used to reach the conclusions drawn in the manuscript and any additional data required to replicate the reported study findings in their entirety. All PLOS journals require that the minimal data set be made fully available. For more information about our data policy, please see <http://journals.plos.org/plosone/s/data-availability>.

Upon re-submitting your revised manuscript, please upload your study’s minimal underlying data set as either Supporting Information files or to a stable, public repository and include the relevant URLs, DOIs, or accession numbers within your revised cover letter. For a list of acceptable repositories, please see <http://journals.plos.org/plosone/s/data-availability#loc-recommended-repositories>. Any potentially identifying patient information must be fully anonymized.

Important: If there are ethical or legal restrictions to sharing your data publicly, please explain these restrictions in detail. Please see our guidelines for more information on what we consider unacceptable restrictions to publicly sharing data: <http://journals.plos.org/plosone/s/data-availability#loc-unacceptable-data-access-restrictions>. Note that it is not acceptable for the authors to be the sole named individuals responsible for ensuring data access.

We will update your Data Availability statement to reflect the information you provide in your cover letter.

**Response:** Done. We have now included the study’s minimal data set as a supplementary file (Table S3) and specified where this minimal data set may be found in the data availability statement.

**Reviewer 1**

The authors present a systematic review with meta-analysis on the relationship between changes in haemoglobin with changes in maximal oxygen consumption. This is an important topic as the ability to carry oxygen in the blood has been shown to influence maximal oxygen consumption. I have a few major concerns that I listed below.

**Response:** Thank you for the detailed review of the manuscript and productive review. The constructive and insightful comments of the reviewer were prudently incorporated into the manuscript and have improved the quality and potential impact of this work.

**1.** I do not believe that it is appropriate to combine interventions simply because they fall under the same category (i.e., all dietary interventions). This would be like combining all pharmacological interventions simply because they are pharmaceuticals. The external validity of these results would be quite limited. Separate analysis for each sub-category would be required to limit clinical heterogeneity.

**Response:** We agree and as suggested by the reviewer, have now added separate analyses for each sub-category. In accordance with Cochrane suggested methodology, a minimum of two studies are needed to perform meta-analyses (please see line 174), limiting several sub-category analyses described below. This approach suggested by the reviewer likely enhances external validity and limits clinical heterogeneity. In an effort of transparent scientific reporting, we also present our original, pooled analyses. For clarity, we have described the categorical separation of categories and sub-categories below.

1. Observational compared to interventional designs. Maintaining this important stratification from the original submission, we continue to separate analyses between observational data and interventional data. In this context, the remaining categories and subcategories are described below.
2. Blood transfusion and blood donation. We have maintained pooled and stratified analyses from the original submission— stratified analyses included 11 studies associated with blood transfusion and 17 studies associated with blood donation. In this context, this section of the text remains unchanged.
3. Erythropoiesis stimulating agents. Our original pooled analysis represented 11 studies examining erythropoietin supplementation and one study examining xenon inhalation. In the revised manuscript, we have stratified our analyses by two different erythropoiesis stimulating agents— erythropoietin and xenon. Please see lines 357-359. Due to an insufficient number of studies (only 1 study), stratified analyses were not performed for xenon inhalation alone.
4. Dietary supplementation. There was a broad spectrum of heterogenous substances used for dietary supplementation. In this context and as now noted in the text (please see lines 378-379), iron and echinacea supplementation were the only sub-categories with an adequate number of studies needed to perform meta-analyses. This important analysis has been added to the text, please see lines 386–392.
5. Environmental hypoxia. Environmental hypoxia analyses pooled two sub-categories— high-altitude environments and normobaric hypoxia. In this framework, the revised manuscript includes analyses stratified into these two sub-categories (high altitude acclimatization and sojourn vs. chronic normobaric hypoxia). Please see lines 409–414.
6. Aerobic (de)training. We have maintained pooled and stratified analyses from the original submission — stratified analyses included 23 studies associated with aerobic training and 7 studies associated with aerobic **de**training. In this context, this section of the text remains unchanged.

**2.** The authors did not describe the differences/limitations in the methods used to measure haemoglobin. The reliability and validity for measures of haemoglobin concentration are very low considering it is strongly affected by changes in plasma volume. Haemoglobin concentration is the gold standard as it is both stable and valid.

**Response:** Thank you for the keen suggestion, we now describe the differences/limitations in the methods used to measure hemoglobin concentration and hemoglobin mass in the main text of the revised manuscript, in both the methods (please see lines 113-125) and the limitations section (please see lines 513-520). Notably, our analyses of the relationship between hemoglobin levels and VO_2_max were performed in duplicate, using both hemoglobin concentration and hemoglobin mass independently. The results of these analyses are highly convergent which provide ecological validity and clinical generalizability to our findings.

**3.** The authors combined studies that included healthy populations with clinical populations. This is not recommended as it may introduce significant clinical heterogeneity. Why did they not conduct a stratified meta-analysis of these groups?

**Response:** The reviewer’s point is well taken and has been incorporated into the revised manuscript, as applicable— for example, please see lines 233-236. Notably, however, articles that reported data for participants with disordered erythropoiesis represented a small portion of the articles included in our analyses— only about 2% (10 of 384 articles). As only a small number of studies enrolled participants with disordered erythropoiesis, it was not plausible to stratify many analyses, specifically among interventional studies. Instead, observational analyses were further stratified for healthy and clinical populations separately (please see lines 257-263).

More broadly, the goal of our work was to examine the relationship between hemoglobin levels and V̇O_2_max, and we intentionally attempted to restrict our analyses to people that may be generally considered as healthy. In this context, our *a priori* methodology specified the inclusion of clinical populations that exhibit alterations in hemoglobin levels without secondary pathologies and which may otherwise be considered as ‘healthy’. As such, the authors carefully screened participants of individual studies and excluded groups of ‘clinical populations’ who had secondary pathologies. This screening process is represented in the PRISMA diagram (**Figure 1**), which shows that 69 studies were excluded due to ineligible participant population.

**4.** I have concerns regarding the analysis of risk of bias of individual studies. I do not believe I have ever seen a systematic review that found that the risk of bias for every category for all intervention studies was ‘low’. Given the large number of studies included in the analysis (158 intervention studies), this would be virtually impossible.

**Response:** The reviewer’s point is well taken and was actively discussed. We acknowledge that the original standards used to assess the risk of bias may have been too lenient. Thus, the risk of bias was reassessed with a more stringent interpretation, and the tables have been updated accordingly (see tables **S1** & **S2**). Risk assessment is now also explicitly described in the text — please see lines 237-247.

However, it is also important to note that our *a priori* approach included rigorous screening and exclusion criteria which resulted in the exclusion of many studies with a probable risk of bias. As evidenced in **Figure 1**, 589 articles were excluded due to ineligible assessments for hemoglobin (386 articles) or VO_2_max values (134 articles) and ineligible participant populations (69 articles). Thus, our *a priori* approach likely contributed to reduced risk of bias among studies included in analyses.

**5.** The statistical methods used to conduct the meta-regressions of the percentage improvement is unclear. Based on the available information, it appears that they used the pooled standard deviation (i.e., combing the baseline standard deviation (SD) with the follow up SD). This would be inappropriate since the SD of the change score is not the same as the pooled SD. The SD of the change score is rarely provided in studies. There are ways to estimate to this value; however, none are described in the methodology.

**Response:** Thank you. We have amended the methods in an effort to improve clarity — please see lines 208-213. We agree with the strong rationale presented by the reviewer:

1) standard deviation would be needed to perform meta-analyses of dependent data regarding the percentage of improvement in VO_2_max.
2) many studies did not report the standard deviation of the percent change in VO_2_max with the applied intervention.

For these reasons, we performed linear regressions (and not meta-regressions) to examine data regarding the percentage of improvement in VO_2_max. Our initial approach was in line with the reviewer’s suggestion, and the methods have been amended to more clearly define this methodology.

**6.** I am unsure how the authors determined the causes of heterogeneity since there are no results that describe that statistical heterogeneity in the manuscript. This not only introduces significant bias in the results but limits the external validity of the findings altogether.

**Response:** Thank you for pointing out this important limitation, and we apologize for the omission in the previous manuscript. We have now added calculations of tau-squared (*τ^2^*) as an estimate of between-study heterogeneity within appropriate findings. Please find amendments to the presentation of results throughout the manuscript.

**Reviewer 2**

I read with great interest and pleasure the article "The relationship between haemoglobin and V̇O_2_max: a systematic review and meta-analysis". The manuscript reflects the magnitude of the task the authors have carried out. From the methodological point of view of a meta-analysis and a systematic review, the manuscript meets the formal and quality conditions usually required for publication in a prestigious journal.

**Response:** Thank you for the many constructive suggestions and supportive tone of the review. These excellent comments have served to improve the quality of this manuscript.

Conducting a systematic review or meta-analysis on the relationship between haemoglobin and VO_2_max is very difficult if practical conclusions are to be derived, because haemoglobin is only one of the possible factors that can limit/determine VO_2_max. Studying the relationship between only one of the determinants and the VO_2_max result has the risk that some readers may not fully understand the real meaning of the relationship or non-relationship of these two variables.

I think it would be advisable to make a minor change, consisting of a more concrete expression of the research question(s), in order to facilitate the understanding of the development of the manuscript and to make the answer(s) to the question(s) more explicitly concrete.

**Response:** Thank you for the keen suggestion. We have amended the abstract and conclusion to enhance the expression of the research question—please see lines 5-10 and 525-528. The overall goal of this work was to provide a comprehensive systematic review and meta-analysis of the relationship between hemoglobin levels and V̇O_2_max in humans from the diverse scientific literature. As the reviewer suggested, there are many putative confounding variables that may limit/determine V̇O_2_max in humans, thus, and we believe this work may offer a foundation to chart future directions and testable hypotheses in this field.

I suggest that the authors, beyond the meta-analysis or systematic review, explicitly state the limitations of the interpretation of the studied relationship:

**Response:** Thank you. We agree and have added a limitations section to the manuscript to highlight the limitations of our simplistic analysis clearly and explicitly— please see lines 496-520.

**a)** The change in haemoglobin in intervention studies, not only involve changes in haemoglobin concentration (mass), but often involve other added changes that can also influence VO_2_max such as a change in blood volume or cardiac output, for example.

**Response:** The reviewer’s point is well taken and was actively discussed. We added a description of some of the potential influence of changes in blood volume and cardiac output in the discussion— please see lines 477-479. Considering the reviewer’s comment, we also further emphasized this topic within newly added limitations section, please see lines 507-512.

**b)** The differences between recipients and donors of blood are not only due to the change in the amount of haemoglobin in the donor or recipient.

**Response:** We agree with the reviewer. Due to the physiological differences in blood donation versus transfusion, stratified analyses were performed. Furthermore, we have amended the discussion to clarify this important point. Please see lines 477-479.

**c)** The summary of the articles used in the systematic review and meta-analysis are based on the interpretation of statistical significance (p-value), but I find no interpretation of the small magnitude of change found in many of the articles reviewed, which are below the minimum detectable change in VO_2_max determination, and well below the minimum clinically significant change. So the changes are less important than apparently suggested or concluded. This point seems to me to be particularly important.

**Response:** Within the interventional analyses, some studies report a large change in VO_2_max, yet others report a small, or ‘negligible’ change in VO_2_max. As the reviewer has mentioned below in comment **d)**, this heterogeneity of findings may be due to differences in duration of the intervention, magnitude of intervention (i.e., dose or amount of blood transfused), or the time of measurement after the end of the intervention. We have added this key concept in the limitations section of the manuscript— please see lines 500-507. Within the meta-analyses stratified by the performed intervention, the pooling of study-level data provides a cumulative finding within the area of interest.

Small, or ‘negligible’ changes in VO_2_max or hemoglobin concentration may be a benefit in the presented regressions by providing a ‘physiological anchor’ to an intervention without physiological alterations. For instance, **Figure 4** denotes the findings from heterogenous studies, some with small changes in VO_2_max and others with large changes in VO_2_max. This heterogeneity may be critical to the performed meta-regressions and linear regressions. Similarly, if all studies showed a large, unidirectional change in hemoglobin levels and VO_2_max, we may not be able to assess a ‘slope’ given that no values near zero would provide directionality.

**d & e)** In the different intervention studies, the changes found are not always comparable according to the duration of the intervention (acute/chronic) or time of measurement after the end of the intervention (hours, days, weeks...).

The methodological limitations of the observational studies used (population variety, methodological variety, ....) are not clearly highlighted.

**Response to d) and e):** These points are well-received. In response to these important considerations, we have added these concepts to a new limitations section. Please see lines 497-512.

**f)** I believe that the conclusions should be improved. If in the introduction they state that there is a consensus on the relationship between haemoglobin and VO_2_ (transport), to what uncertainties in knowledge on the subject did the authors intend to respond with their review and meta-analysis?

**Response:** This work aimed to provide a comprehensive review of the diverse scientific corpus. One example of uncertainty is presented in lines 62-68. Moreover, this work may help identify further areas of uncertainty and stimulate research to better our understanding of the physiological consequences of interventional manipulation of hemoglobin levels. By creating an unbiased synthesis of this relationship (between hemoglobin levels and V̇O_2_max in humans), we believe our work may serve as a foundation to chart future directions and testable hypotheses in this field. The conclusion has been amended to better describe the aim of this work. Please see lines 525-528.

**g)** I believe that it does not follow from their study that the improvement in performance (they have not studied it) caused by the increase in haemoglobin is an argument for the prohibition of blood doping. It should be banned because it is an artificial method, because it is a potentially very dangerous method, and because it increases performance, not necessarily because of the increase in haemoglobin.

**Response:** Thank you for your comment. We have removed this sentence.

**h)** Finally, I believe that the bibliographical references are abused, and there are a good number of them that could be removed without altering the quality of the study. For example, I do not think it is necessary to use 6 articles to support a clear statement (lin 42), one would be sufficient. Furthermore, some references are not very relevant in the context in which they have been used, such as ref. 23, which does not study the VO_2_-Hb ratio, but is a review of anaemia in athletes (lin 47). I also fail to understand the use of reference 35 to justify that above 500m altitude can be considered a hypoxic-hypobaric environment, nor that FIO2 is less than 20%, nor do I agree with that statement, nor do I believe that such a statement follows from reference 35.

**Response:** Our apologies, we certainly understand the reviewer’s point. Although our intention was to pay homage to the many luminaries in this field, we understand how this was construed as abusing bibliographical references. In this context, we have removed several citations in the introduction (*n*=8) and discussion (*n*=6). Additionally, we have revised the methodology to more accurately denote the environmental conditions eligible for the determination of VO_2_max. Please see lines 110-112.

I submit these observations for your consideration, which I believe will make your manuscript more useful, and I hope they will be addressed (if you deem it appropriate) or responded to in a reasoned manner.

In any case, I would like to congratulate you on your work.

**Response:** Many thanks. The comments from the reviewers represent a significant investment of time and effort, and we believe the comments enhanced the quality and potential impact of this manuscript.
