# Supplementary material for: The relationship between hemoglobin and V˙O2max: A systematic review and meta-analysis
Source: PLoS One. 2023 Oct 12;18(10):e0292835. doi: 10.1371/journal.pone.0292835 (PMC10569622; doi:10.1371/journal.pone.0292835)
Supplement: Attachment — Submitted filename: PONE-D-22-29015R2-R2R2S1.docx [file pone.0292835.s008.docx]

**Academic Editor**

Thank you for submitting your manuscript to PLOS ONE. After careful consideration, we feel that it has merit but does not fully meet PLOS ONE’s publication criteria as it currently stands. Therefore, we invite you to submit a revised version of the manuscript that addresses the points raised during the review process.

**Response:** Thank you for spearheading this high-quality, collegial, and productive review process. As we described previously, the comments from the reviewers enhanced the quality and potential impact of this manuscript.

Please, address, the best you can, the reviewers' suggestions. He is a true expert on this topic and on the methodology of a systematic review.

**Response:** We read with great interest the comments of the reviewer and thank them for the efforts to improve our manuscript. We appreciate the many constructive suggestions.

**Reviewer 1**

I would like to start by commending the authors for addressing my initial feedback. They have addressed significant methodological issues. Below I comment on several specific issues that should help to improve the overall quality of their review.

**Response:** Although not the intent, we recognize the following comments may be interpreted as gratuitously flattering. We would like to thank the reviewer for the thorough and constructive review of this manuscript. The comments from the reviewer represent a significant investment of time and effort, and we believe the comments enhanced the quality and potential impact of this manuscript. Thank you.

Please also note that throughout the response to reviewers, specific text excerpts are referenced using line numbers— these line numbers are associated with the redlined version of the manuscript.

**Comment 1** | Please use the headings listed in the PRISMA checklist. Further, there are sub-headings that should be used to organize each of the sections of the review. This will improve both the transparency and flow.

Details with a full guidance is available in the second reference below.

**References**

Page MJ, McKenzie JE, Bossuyt PM, Boutron I, Hoffmann TC, Mulrow CD, et al. The PRISMA 2020 statement: an updated guideline for reporting systematic reviews. BMJ. 2021;372:n71.

Page MJ, Moher D, Bossuyt PM, Boutron I, Hoffmann TC, Mulrow CD, et al. PRISMA 2020 explanation and elaboration: updated guidance and exemplars for reporting systematic reviews. BMJ. 2021;372:n160.

**Response:** Done. We have changed the headings (Level 1 Headings) and sub-headings (Level 2 Headings) to align with the headings and sub-headings associated with the PRISMA checklist. Please find these new headings informed by the PRISMA checklist throughout the manuscript. For additional organization for readers, we have also provided Level 3 Headings as appropriate. Notably, however, the PRISMA checklist does not (explicitly) offer suggestions for Level 3 Headings. In this context, we discussed Level 3 Headings intensively within our author team and included headings as warranted.

**INTRODUCTION**

**Comment 2 | Page 4, line 39:** It is important to describe how a VO2max is assessed and what physiological measurements constitutes a VO2max. It would also be beneficial to explain the difference between VO2max and VO2peak. I am assuming that the authors combined studies that included both measurements.

**Response:** Thank you for pointing out these keen points. We have included additional contextual background on V̇O_2_max testing within the manuscript (please see **lines 110 to 115**) and responded to your comments below.

We have considered your comment as two separate points. **First,** the standardized assessment of V̇O_2_max is described in the methods under the “Eligibility criteria” sub-heading— please see **lines 102 to 122**. We have added additional text to clarify the criteria used to qualify measurements as V̇O_2_max (as opposed to V̇O_2_peak).

**Second**, as also described in the methods under the “Eligibility criteria sub-heading”, the authors — in accordance with our *a priori* protocol — discussed the inclusion of V̇O_2_peak measurements intensively before data collection commenced and decided to only include measurements that represent V̇O_2_max. In this framework, studies that assessed V̇O_2_peak or *estimated* V̇O_2_max were not eligible for inclusion.

**Comment 3 | Page 4, line 50:** This section should be removed from the introduction. It may be more appropriate in the methods section to explain the eligibility criteria, or more importantly in the limitations section of the review.

**Comment 4 | Page 4, line 60:** “Although a strong …”. If we already know that there is a strong relationship between haemoglobin levels and VO2max, why conduct this review? Maybe acknowledge all the work that has been done in the past then go right into the lack or potential lack of systematic reviews with meta-analysis. The authors are on the right track by need to strengthen their rationale. It may also be beneficial to acknowledge previous systematic reviews on the topic, some of the limitations in those reviews, and how this review differs (e.g., addresses previous limitations, looks at other outcomes or interventions).

**Response:** We have elected to respond to comments 3 and 4 from the reviewer – which both correspond to the same section of the manuscript—using one response. We appreciate the reviewer’s perspectives and have more aptly entitled this section of the manuscript as “Rationale”. In accordance with the description of the rationale section provided in the PRISMA checklist – describe the rationale for the review in the context of existing knowledge – we have described our primary rationale within the context of existing knowledge. While we agree with the reviewer that this information may also be appropriate in the methods or limitations section, this information describes the rationale for the review and continues to be within the introduction section.

As described in the text, although many studies have been performed on the relationship between hemoglobin levels and V̇O_2_max, there are ambiguous areas associated with biological diversity and heterogenous study designs, and a comprehensive synthesis of information may provide key insights from the scientific corpus.

**Comment 5 | Page 5, line 74:** “Moreover…” This is a secondary objective. The authors use both specific (sex) and general (interventions) examples. First, it is important to state that there is a secondary objective. Second, it may be better to explain that you will conduct secondary analyses to determine the influence of ‘population characteristics’ (sex, age, etc.) and ‘intervention characteristics’. Specific examples of stratified and meta-regression should be provided in the Methods (i.e., how you will address statistical heterogeneity).

**Response:** Done. We agree and have added explicit acknowledgement that there is a secondary objective. Additionally, we included that the secondary analyses were conducted to determine the influence of population characteristics and intervention characteristics—both with inclusion of specific examples. Please find this revised section on **lines 76 to 79**.

**METHODS**

**Comment 6 |** Please list and describe effect measures that will be used to describe the results. I believe that some of this data is in the “Statistical analysis” section. This should be in a section called “Effect Measures”.

**Response:** Done. As requested, we have listed and described effect measures that are used to describe the results. This important information may now be found under the “Effect measures” subheading—please see **lines 181 to 187**.

**Comment 7 |** Have you considered using p-values to calculate the missing standard deviation by using a dependent t-test? This could allow you to conduct meta-regressions (albeit a more conservative analysis when p-values or only listed as p < 0.05). I would consider a p-value of “<0.05” as p = 0.0499 if using a t-test to calculate a SD, though any assumptions should be listed.

**Response:** We did consider this important point from the reviewer. However, no paper provided means and p-values without estimate of error. In this context, this suggested data transformation was not warranted. Please find that we have removed this section from the text—see **lines 159 to 161**.

**Comment 8 |** All regressions that do not consider the within and between group variance must be removed from the results. It is not appropriate to pool studies without considering the weight of each study as the results will mislead the reader. Practitioners use meta-analyses to make decisions about patient care so they must be conducted accurately.

**Response:** Thank you for the comment. We agree with the reviewer that there are limitations in using regressions that do not consider within group variance and between group variance. Within this manuscript, all regressions performed considered between group variance. However, applied linear regressions do not account for within group variance due to each data point considered as a mean without measurement of standard error. The linear regressions performed are limited by the available data and this limitation has been appropriately caveated within the methods (please see **lines 226 to 233**). When transforming the data to examine the effects of manipulating hemoglobin levels on changes in V̇O_2_max, no manuscripts report the percent change with associated standard error. We have added this topic as a limitation in the discussion (please see **lines 524 to 542**). Although these linear regressions have limitations, we have elected to keep these analyses as they provide an important comparison with other seminal work in the field [1,2].

**[1]** Calbet JA, Lundby C, Koskolou M, Boushel R. Importance of hemoglobin concentration to exercise: acute manipulations. Respir Physiol Neurobiol. 2006 Apr 28;151(2-3):132-40. doi: 10.1016/j.resp.2006.01.014. Epub 2006 Mar 3. PMID: 16516566.

**[2]** Ekblom BT. Blood boosting and sport. Baillieres Best Pract Res Clin Endocrinol Metab. 2000 Mar;14(1):89-98. doi: 10.1053/beem.2000.0056. PMID: 10932813.

**Comment 9 | Page 6, line 88:** Why was only a single database used to conduct the literature search? Are you concerned that you have important missed studies?

**Response:** During our preliminary literature searches, we were deluged with high quality scientific articles. During preliminary searches, we also found significant duplication between databases. Thus, after preliminary searches, we elected to use a single database to conduct this literature search. This important limitation has been added to the limitations section – please see **lines 524 to 525**. After carefully searching references of all included articles for additional inclusions, we only found three additional studies to include. In this context, we are not concerned that we have important missed studies, and we were very pleased to have meta-analyzed data from nearly 400 studies.

**Comment 10 | Page 8, line 149:** Why was data that included confidence intervals as a measure of uncertainty excluded when transformations can easily be performed, especially since this was does for SEM to SD.

**Response:** Thank you for this comment. We looked back through our exclusion criteria for each study and found that no study was excluded on the basis of confidence intervals as a measure of uncertainty. Thus, we have removed this excerpt from the manuscript— please find strike-through text on **lines 159 to 161**.

**Comment 11 | Page 8, line 149:** If data points that were only expressed as means without associated error, why were studies included in a non-weighted regression?

**Response:** Good point. As discussed in response to **Comment 8**, we did not include data points that were expressed as means without associated error in meta-regression analyses. However, applied linear regressions were performed and presented using means without associated error, and these analyses have been appropriately caveated throughout the text.

**Comment 12 | Page 8, line 151:** This section should be in the section ‘Data Items’ as described in PRISMA. The items should follow PICOS.

**Response:** These changes have been made as requested by the reviewer, and the section entitled ‘Data Items’ follows PICOS. Please see **lines 143 to 161**.

**Comment 13 | Page 9, line 169:** Statistical Analysis should be divided into Effect Measures, Synthesis Methods, and Reporting Bias Assessment, as indicated by PRISMA.

**Response:** Done. As suggested by the reviewer and indicated by PRISMA, the statistical analysis section has been divided into Effect Measures, Synthesis Methods, and Reporting Bias Assessment. Please see **lines 172 to 233**.

**Comment 14 | Page 9, line 173:** “Analyses pooled outcome data…”. This sentence does not make sense. The analyses didn’t pool the data, the authors did. Second, it is stated that data was pooled from each study but the sentence before states that they were pooled by study design.

**Response:** In line with these suggestions from the reviewer, we have clarified this section. Please see **line 192**.

**Comment 15** | I think sensitivity analyses should be performed for: 1. Relationship for haemoglobin with VO2peak vs VO2max, 2. Study design.

**Response:** Thank you for these suggested analyses. First, this review only included studies that assessed V̇O_2_max, and thus excluded studies that assessed V̇O_2_peak. In this framework, the requested sensitivity analyses for the relationship for hemoglobin with V̇O_2_peak vs V̇O_2_max is beyond the *a priori* scope of the review. Second, stratified/subgroup analyses between study design are included in the review. From our perspective, there were no decision nodes within this systematic review process that generated a need for a sensitivity analysis — the sequence of decisions for inclusion were clearly objective and non-contentious [1].

**[1]** Cochrane Handbook for Systematic Reviews of Interventions, Part 2: General methods for Cochrane reviews, Chapter 9: Analysing data and undertaking meta-analyses, Section 9.7 Sensitivity analyses.

**Comment 16 | Page 10, line 176:** Can you please use Higgin’s I^2 statistic to report the statistical heterogeneity. It does have limitations (as does Q and tau^2), but it is a measure that explains the relative statistical heterogeneity and is also easier for the reader to understand (especially those that do not understand meta-analysis).

**Response:** Done. As suggested by the reviewer, the Higgin’s I^2^ statistic has been added to report the relative statistical heterogeneity of meta-analyses within the review.

**Comment 17 |** How did you account for small sample bias or publication bias? I would suggest Egger’s for small sample size and Vevea and Hedges Weight-Function Model for Publication Bias.

**Response:** In line with the suggestion by the reviewer, Egger’s test was used to assess potential publication bias in meta-analyses [1]. This has been added as a method (please see **lines 179 to 180**) and the results of these tests are presented in the text (please see **lines 266 to 271**).

**[1]** https://training.cochrane.org/resource/identifying-publication-bias-meta-analyses-continuous-outcomes

RESULTS

**Comment 18 |** All results should be described in past tense.

**Response:** Thank you for this suggestion. Generally, we agree and have assured that results are described in past tense. As an important caveat, there are several atemporal facts which, according to standardized grammatical rules, are presented using present tense. These atemporal factors are primarily associated with presentation of results in the tables and figures of the manuscript, which exist without relation to time. As an example, from **lines 236 to 237**— “The process of study selection and article categorization is represented…[in Fig 1]”

**Comment 19 |** Please use the PRISMA headings as mentioned above.

**Response:** Thank you for this suggestion. As described in response to **Comment 1** above, PRISMA checklist has informed Level 1 and Level 2 Headings throughout the manuscript.

**Comment 20 |** If stratified analyses were performed (e.g., by sex: combined, male, female), please provide results that describe if there were significance (p-value) differences for each analysis (either from the initial analysis or between (among) stratified groups. This should be completed for all results in text and in tables.

**Response:** Done. As described on **lines 293 to 295**, exploratory analyses stratifying meta-regression models by biological sex found no sex differences in the relationship between hemoglobin mass and V̇O_2_max values. In addition to describing if there were significant differences for each analysis, we have provided adequate information for the interested reader to perform additional analyses or impute findings.

**Comment 21 | Page 16, line 314:** Why was the study removed? Was it the ‘only’ one study? This could provide a rationale for the removal, but needs to be explained to the reader.

**Response:** The study referenced by the reviewer represents a study of a ‘clinical population’. This study was removed in response to the first round of revisions and concern about combining studies that included healthy populations with clinical populations. Indeed, it was ‘only’ one study removed as appropriately described in the text. Rationale for the removal has now been explicitly stated for the reader— please see **lines 339 to 342**.

DISCUSSION

**Comment 24 | General:** Please use or at least try to address PRISMA sub-headings. 1. General interpretation of results, 2. Limitations of evidence included in the review, 3. Limitations of review process, 4. Implications for practice, policy and future research.

**Response:** As requested, we have addressed the PRISMA sub-headings within the text, including:

1. General interpretation of results (paragraph 1 of discussion),
2. Limitations of evidence included in the review (limitations section of discussion),
3. Limitations of review process (limitations section of discussion),
4. Implications for practice, policy, and future research (ultimate paragraph of discussion entitled ‘conclusion’).

**Comment 25 | Page 24, line 497:** What do you mean by simplistic design?

**Response:** Because we limited our focus to a single relationship between hemoglobin and V̇O_2_max, our designed approach did not consider the potential confounding effects of other relevant physiological factors, such as blood volume, plasma volume, maximum cardiac output, and muscle oxygen diffusivity. In this context, our primary analyses were simplistic — our analyses treated a complex biological relation as if it were much simple than it is. This section has been clarified—please see **lines 525 to 530**.

**Comment 26 | Page 24, line 499:** What are you referring to by “more complex statistical methods”? You can use aggregate data to perform multivariate analyses. Limitations in the complexity of statistical analyses are probably due to the software that used to conduct the analyses. Software packages available in ‘R’ make it very easy to conduct multivariate analyses. The primary (and albeit significant) limitation using aggregate data is the fallacy of regression to the mean, which occurs when using continuous data of population characteristics to perform meta-regressions. However, this limitation occurs regardless of the type of analyses (i.e., univariate or multivariate analyses).

**Comment 27 | Page 24, line 504:** “For example…” This point is only a limitation if you choose not to run stratified or meta-regressions on intervention characteristics, which were completed for some variables. You can say that the limitations, specifically with your review was that you chose not to run these analyses for which there may be valid reasons which should be stated (e.g., the data was not available, etc.). But I would explain why you chose not examine the variables listed here. However, it is not a limitation to the approach as it is best to include all studies and then find out the source of statistical heterogeneity by means of the clinical heterogeneity.

**Response:** We have elected to respond to comments 26 and 27 from the reviewer – which both correspond to the same section of the manuscript—using one response. We agree with these sage remarks from the reviewer. We have removed the phrase ‘more complex statistical methods’, and more explicitly described the limitations of our approach. Please find the revised section on **lines 524 to 550**.

**Comment 28 | Page 25, line 513:** Good point. Though the primary differences in haemoglobin would be concentration versus mass. Please cite the literature for your examples. If you believe that your results show that there was no difference between the two measurement types, please explain this to the reader in further detail as it is an important finding that conflicts with current literature.

**Response:** Done. We have cited the literature for our examples, please find this citation both in the methods (**line 129**) and limitations section of the discussion (**line 547**). We do not believe that there is no difference between the two measurement types and our analyses did not evaluate the potential difference between the two measurement types.

**CONCLUSION**

**Comment 29 | Page 25, line 522:** The results suggest that the pooled effect measure changed after addressing measures of clinical heterogeneity (though, it was not stated in the results section of the differences in modifiers were considered to be significant (no p-value to accompany the change in variance). Therefore, given the available results, I am not sure if this statement is true.

**Response:** Point well taken. We have removed the phrase “across a heterogeneous pool of studies”, and the concluding statement is now true without ambiguity.

**Comment 30 | Page 25, line 525:** I would argue that there is still bias in the methods used to conduct the synthesis of the results. However, there are efforts within the manuscript to limit the degree of bias.

**Response:** We agree. The term “unbiased” has been removed and replaced with “comprehensive”.

**Reviewer 2**

I have reviewed the document submitted by the authors again and have observed that the changes made in this second manuscript are minimal compared to the first, except for some statistical treatments (heterogeneity, Tau). The observations made in the first round are mainly included in a broad section of limitations that have been added to this second document.

The fundamental objections remain present in this second manuscript. Nevertheless, considering the magnitude of the effort made, the number of articles reviewed, and the fact that the limitations of the study are now explicitly stated based on the observations made, I believe that the article can be published. I think it is sufficiently clear what the real scope of the work's conclusions may be for non-expert readers in the field.

**Response:** Thank you for the review of the manuscript. We agree that the limitations section has provided additional and relevant context for the appropriate interpretation of these data.
