# Supplementary material for: The relationship between hemoglobin and V˙O2max: A systematic review and meta-analysis
Source: PLoS One. 2023 Oct 12;18(10):e0292835. doi: 10.1371/journal.pone.0292835 (PMC10569622; doi:10.1371/journal.pone.0292835)
Supplement: Attachment — Submitted filename: PONE-D-22-29015-R2R3S1.docx [file pone.0292835.s009.docx]

**Academic Editor**

Thank you for submitting your manuscript to PLOS ONE. After careful consideration, we feel that it has merit but does not fully meet PLOS ONE’s publication criteria as it currently stands. Therefore, we invite you to submit a revised version of the manuscript that addresses the points raised during the review process.

I have asked a statistician to assist us because of the concerns raised by the reviewer. The comments would seem minor to me but feel free to address the best you can, in an efficient manner, the changes requested. If needed, you can also include supplementary data to save space in the main manuscript. Sorry for this but we hope you understand we all are working to publish the best possible article.

**Response:** As described previously, thank you for spearheading this review process, which has augmented the quality and potential impact of this manuscript.

**Reviewer 1**

The authors have done a great job addressing the feedback that I provided. They have made significant improvements to the quality of their manuscript.

**Response:** We thank the reviewer for the thorough and constructive reviews of the manuscript.

The only issue remaining is that all linear regressions must be removed from the analysis as they do not consider both within and between study variance. A linear regression can be used for individual patient data sets. However, it is not appropriate for aggregate data. If the measure of uncertainty (i.e., standard error) is unavailable then the data cannot not be pooled. The authors indicate that these analyses provide an important comparison to other work, which may be true. However, the methods used to conduct the analyses are incorrect.

**Response:** All linear regressions have been removed from the manuscript.

**Reviewer 3**

The present is a high quality paper. Only minor issues

**Response:** Thank you for the review of the manuscript and supportive comment.

Abstract: it may be worth to have only one primary end point

**Response:** Indeed, we do have one primary end point. As described in the ‘objectives’ section of the manuscript (please see lines 74 to 79), the primary end point of this systematic review and meta-analysis aimed to evaluate the relationship between hemoglobin levels and V̇O_2max_. Because both hemoglobin levels and V̇O_2max_ may be described using multiple metrics, our primary end point is associated with several different analyses.

methods: it should be added hiow many authors performed the search

**Response:** This important information has been added—please see lines 92 to 93.

methods: it should be added if random or fixed effect was used

**Response:** We used random-effects models—please see lines 187 to 189.

methods; subgroup analysis according to kind of studies should be added

**Response:** Indeed, as a secondary objective, prespecified subgroup analyses were performed according to the kind of study. Separate analyses were included for observational studies and interventional studies, and additional analyses performed based on intervention (blood transfusion or donation, erythropoiesis stimulating agents, dietary supplementation, environmental hypoxia, or aerobic (de)training). We have revised the methods to more clearly delineate the subgroup analyses – please see lines 215 to 217.
